# Mentes ativas (active minds): effects of a remote cognitive stimulation program

**DOI:** 10.1590/1980-5764-DN-2025-0397

**Published:** 2026-06-15

**Authors:** Sabrina Aparecida da Silva, Gabriela dos Santos, Tiago Nascimento Ordonez, Maria Antônia Antunes de Souza, Diana dos Santos Bacelar, Yasmin dos Santos Rodrigues, Bruna Magro Fioramonte, Beatriz Aparecida Ozello Gutierrez, Thais Bento Lima da Silva

**Affiliations:** 1 Universidade de São Paulo, Escola de Artes, Ciências e Humanidades, Pós-Graduação em Gerontologia, São Paulo SP, Brazil.; 2 Universidade Federal do Recôncavo da Bahia, Centro de Ciências da Saúde, Santo Antônio de Jesus BA, Brazil.; 3 Universidade de São Paulo, Hospital das Clínicas, Grupo de Neurologia Cognitiva e do Comportamento, São Paulo SP, Brazil.

**Keywords:** Cognition, Aged, Aging, Health Promotion, Cognição, Idoso, Envelhecimento, Promoção da Saúde

## Abstract

With population aging, the prevalence of cognitive deficits increases, highlighting the importance of nonpharmacological interventions for promoting mental health in older adults.

**Objective:**

To evaluate the efficacy of a remote cognitive stimulation program (*Mentes Ativas*) in cognitively healthy older adults.

**Methods:**

The initial sample included 56 participants enrolled in the USP 60+ program at the School of Arts, Sciences, and Humanities of the University of São Paulo. For the final analyses, 22 participants who completed at least 80% of the sessions over a six-month period were assessed. Assessments included the Geriatric Depression Scale, Geriatric Anxiety Inventory, Cognitive Function Instrument, and Satisfaction with Life Scale. Data were collected at baseline and post-intervention and analyzed using non-parametric statistical tests.

**Results:**

There was a significant reduction in perceived subjective cognitive decline (mean score from 4.07 to 3.07; p=0.005) and in depressive symptoms (3.55 to 2.32; p=0.046), as well as a decrease in anxiety symptoms (5.88 to 3.95) and slight improvement in life satisfaction (7.59 to 7.95), although not statistically significant (p=0.132). Significant correlations were found among greater perceived cognitive decline, both depressive and anxiety symptoms, and with lower life satisfaction.

**Conclusion:**

The *Mentes Ativas* program may help promote cognitive and psychosocial improvements in older adults, reinforcing the role of cognitive interventions in promoting healthy aging.

## RESUMO

Com o envelhecimento populacional, aumenta a prevalência de déficits cognitivos, ressaltando a importância de intervenções não farmacológicas para a promoção da saúde mental em pessoas idosas.

**Objetivo:**

Avaliar a eficácia de um programa de estimulação cognitiva remoto em pessoas idosas cognitivamente saudáveis.

**Métodos:**

A amostra inicial contou com 56 participantes, inscritos no programa USP 60+ da Escola de Artes, Ciências e Humanidades da Universidade de São Paulo. Para as análises finais, foram considerados 22 participantes que completaram pelo menos 80% das sessões ao longo de seis meses. As avaliações incluíram a Escala de Depressão Geriátrica, o Inventário de Ansiedade Geriátrica, o Instrumento de Função Cognitiva e a Escala de Satisfação Geral com a Vida. Os dados foram coletados no início e ao final da intervenção e analisados por testes estatísticos não paramétricos.

**Resultados:**

Houve redução significativa na percepção subjetiva de declínio cognitivo (pontuação média de 4,07 para 3,07; p=0,005) e nos sintomas depressivos (3,55 para 2,32; p=0,046), além de diminuição dos sintomas ansiosos (5,88 para 3,95) e leve melhora na satisfação com a vida (7,59 para 7,95), sem significância estatística (p=0,132). Foram encontradas correlações significativas entre maior percepção de declínio cognitivo, sintomas depressivos e ansiosos e menor satisfação com a vida.

**Conclusão:**

O programa “Mentes Ativas” pode contribuir para a melhora cognitiva e psicossocial em pessoas idosas, reforçando a relevância das intervenções cognitivas na promoção do envelhecimento saudável.

## INTRODUCTION

 Population aging, driven by greater life expectancy and lower birth rate, have led to a significant increase in the proportion of older people. This phenomenon is associated with a higher prevalence of neurodegenerative diseases, especially dementia, negatively impacting the autonomy and functioning of this population^1,2^. Despite advancements in diagnosis and pharmacological interventions for Alzheimer’s disease, no cure is currently available, underscoring the importance of non-pharmacological strategies, such as cognitive stimulation, to mitigate cognitive decline and help maintain quality of life (QoL)^3,4^ . 

 The cognitive reserve theory holds that greater brain activity and engagement in cognitively stimulating activities can delay the onset of cognitive deficits^5^ . Evidence from systematic reviews and meta-analyses show that cognitive stimulation interventions promote significant improvement in cognitive functions, wellbeing and social interaction^3,6,7^. 

 Subjective cognitive decline (SCD) is among the first signs of cognitive impairment, characterized by self-reported perception of worsening in memory or other cognitive functions, even though performance on neuropsychological tests remains within normal range. SCD has been identified as a potential early marker for the development of mild cognitive impairment (MCI) and dementia, and is also associated with depressive symptoms and reduced QoL^8^ . 

 Of the different modes of intervention for preventing or minimizing the forms of cognitive impairment, working memory (WM) training stands out. WM is an essential cognitive function of the short-term memory system and responsible for simultaneous storage and processing of information^9,10^. Studies suggest that interventions focused on WM are effective for maintaining or enhancing cognitive performance in healthy older adults^9,11,12^ . 

 With advances in digital technologies and the need to adapt services in response to the COVID-19 pandemic, interest in remote cognitive stimulation programs has grown. Studies show that online interventions are feasible and can have similar efficacy to face-to-face modes of delivery, while offering greater accessibility and flexibility for older adults with different sociodemographic profiles^13-15^. 

 Remote cognitive interventions have several major advantages, such as reducing geographic barriers, decreasing costs associated with travel, and allowing greater participant adherence, particularly for individuals with mobility issues or who reside in rural areas. Moreover, the use of digital platforms enables personalization of activities and continuous monitoring of participant progress, increasing the efficacy potential of these interventions^15^ . 

 However, challenges involving familiarity with technology, internet access, and technical support continue to represent significant barriers to implementing remote programs, especially in underserved populations. Hence, it is vital that programs be tailored to the specific abilities and needs of the older population, including prior training on the use of technology and personalized monitoring during interventions^13^ . 

 There has been a growing number of investigations on cognitive interventions for older adults with different cognitive performance profiles. In the absence of curative drug-based strategies for dementia, cognitive interventions are becoming increasingly recognized as an important strategy for maintaining cognition. Cognitive interventions have proven to be a safe, lowcost approach without adverse effects for the older population seeking to prevent or mitigate cognitive impairment. Therefore, set against the challenges and opportunities associated with implementing remote cognitive interventions, the objective of the present study was to assess the efficacy of an online cognitive stimulation program in cognitively healthy older adults, along with its impact on cognitive functions and psychosocial variables. 

## METHODS

### Participants

 The sample for the initial collection at baseline (T0) comprised 56 older adults from São Paulo city, enrolled on the Open University Program — USP 60+ at the School of Arts, Sciences, and Humanities of the University of São Paulo (EACH-USP). The USP 60+ program offers initiatives that support biological, psychological and social aspects of community-dwelling older adults, integrating teaching, research and university outreach activities. 

### Inclusion criteria

 The study included individuals aged ≥60 years, with education ≥4 years, and cognitive performance of above 15 points on the Brazilian telephone-based Mini-Mental State Exam (MMSE-BRAZTEL)^16^ . Other criteria for inclusion were a score of less than 6 on the Geriatric Depression Scale (GDS)^17^ and less than 10 on the Geriatric Anxiety Inventory (GAI)^18^ . Participants also had to have access to a device with internet connection and basic working knowledge on using the Google Meet platform. The presence of controlled chronic diseases was not considered a criterion for non-inclusion. 

### Exclusion criteria

 Individuals who had taken part in cognitive training within the last 12 months, exhibited signs of dementia, presented major depressive or anxiety disorders, and functional deficit, were excluded. Inclusion and exclusion criteria were checked using scales or self-reports. Only participants (22 older adults) with an 80% attendance rate of intervention sessions were included in the analyses. 

### Sociodemographic variables

 A structured questionnaire was used to collect sociodemographic and health status information from the study participants. Socioeconomic level was estimated based on the Brazilian Economic Classification Criteria of the Brazilian Association of Market Research Institutes^19^ . Professional occupation was defined as the main activity performed during the participant’s working life. 

### Psychosocial variables

 Psychosocial variables were assessed using previously validated instruments applied to the Brazilian older population. Depressive symptoms were investigated using the 15-item GDS (GDS-15), a questionnaire containing dichotomous responses, culturally adapted for use in Brazil. The scale includes somatic components and is suitable for screening depression in older adults, having been previously validated for international diagnostic criteria^17^ . 

 Symptoms of anxiety were assessed using the GAI, comprising 20 statements which the participants rate as true or not. The final score on the GAI ranged from 0 to 20, with scores above 10 or 11 indicating a higher probability of anxious disorders^18^ . 

 Perceived subjective cognitive impairment was measured using the Cognitive Function Instrument (CFI)^20^ , which probes perceived changes in cognition over the past year. Response options are Yes, No or Maybe, with scores above 2.5 suggesting the presence of subjective cognitive decline. 

 Lastly, subjective well-being was measured as a single item using a visual scale from 1 to 10, on which participants rated their overall satisfaction with life, with a value of 1 representing the worst living conditions imaginable and 10 the best conditions^21^ . 

### Procedures

 The cognitive assessment was administered by undergraduates from the Gerontology degree program of the EACH-USP, after having received around 45 minutes of training by the researchers. All participants signed the Informed Consent Form, assuring participants anonymity, the confidentiality of information provided, and the right to discontinue participation at any time. 

 Participant recruitment took place during the second semester of 2024. The sample was assessed at two time points: program start (baseline — T0) and program end (+6 months — T1). The cognitive training protocol (Chart 1)2^22^ included an educational component on memory and aging, practical activities with cognitive tasks to implement compensatory strategies, and actions to generalize these strategies to activities of daily living. 

**Chart 1:** Model of activities performed in cognitive stimulation sessions of the Mentes Ativas program^22^ .

Session	Ability	Activity
1st	Creativity and attention	Game — “Pass it on or what’s the question?”: from a card that contains the answer to a question, players must try to guess what the original question was that prompted the answer.
	Attention, concentration and working memory	- Brain warm-up: 1) Think of a word that contains all the vowels; 2) Share the words thought of; 3) List as many words as possible in reverse alphabetical order. - Playing: Musical notes corresponding to numbers are played and, upon hearing prompts, students must perform the calculation on the abacus. - Individual training
	Logical reasoning and visuospatial ability	Challenge of the week: - Calcudoku: fill the squares with the missing numbers which, when summed, give the result required.
	Language and attention	Game — “Scrambled words”, based on a category, students must choose a word, write it in a scrambled form, and show it to the class, who must then try to guess the original word.

### Statistical analyses

 The statistical analyses were designed to describe the sample profile, investigate pre- and post-intervention differences, and explore associations among sociodemographic, cognitive and psychosocial variables. Firstly, participants were characterized using descriptive statistics (mean, standard deviation, minimum, maximum and median) and relative frequencies. 

 Prior to electing the inferential tests, the Shapiro-Wilk test was applied to determine whether the data distribution met the assumption of normality. The results showed absence of a normal distribution for the variables analyzed. Consequently, non-parametric tests were employed for subsequent analyses. 

 To assess potential attrition bias, the Mann-Whitney U test was used to compare continuous variables between independent groups (Completers vs. Dropouts) at baseline, while the χ^2^ test was utilized for categorical variables. The Wilcoxon test for paired samples was used to compare scores at time points T0 (pre-test) and T1 (post-test), given its suitability for paired data that do not follow a normal distribution. Spearman’s correlation coefficient was used to investigate associations between continuous and ordinal variables, being suitable for small samples and non-parametric data. A significance level of 5% (p<0.05) was adopted for all statistical analyses. 

## RESULTS

 The sample comprised 56 participants with a mean age of 68.21 (standard deviation — SD=5.98) years, and were predominantly female (80.36%). Regarding marital status, most participants were married or in a civil union (48.21%), followed by separated/divorced (25%). The educational level of participants was high, where 80.36% had completed higher education, and mean time engaged in formal study was 17.59 (SD=7.33) years. The majority of participants were retired or pensioners (94.64%). With respect to living arrangements, family compositions varied, with most participants living with a partner (35.71%) or alone (32.14%). These data reveal a relatively high level of education and autonomy, factors which may have positively influenced intervention adherence and results (Table 1). 

**Table 1:** Sociodemographic variables

Variables			n	%	Mean	SD	Minimum	Median	Maximum
Age (years)			56	100.00	68.21	5.98	60.00	67.00	86.00
	Sex	Female	45	80.36					
		Male	11	19.64					
	Marital status	Widowed	8	14.29					
		Married/civil union	27	48.21					
		Separated/divorced	14	25.00					
		Single	7	12.50					
	Education	Primary complete	1	1.79					
		Secondary incomplete	1	1.79					
		Secondary complete	4	7.14					
		Higher incomplete	5	8.93					
		Higher complete	45	80.36					
Education (years)			56	100.00	17.59	7.33	2.00	17.00	58.00
	Retired or pensioner?	Yes	53	94.64					
		No	3	5.36					
	Who do you live with?	Alone	18	32.14					
		With partner only	20	35.71					
		With partner and your children	7	12.50					
		With your children	7	12.50					
		Your children live with you	2	3.57					
		Others live with you	1	1.79					
		With partner, children and grandchildren	1	1.79					

Abbreviation: SD, standard deviation.

 To assess potential selection bias due to attrition, baseline characteristics (T0) were compared between participants who completed the intervention (Completers, n=22) and those who dropped out or did not meet the attendance criteria (Dropouts, n=34). As shown in Table 2 and Table 3, there were no statistically significant differences between the groups regarding sociodemographic variables or baseline clinical scores (CFI, GDS, GAI, and Satisfaction With Life Scale — SWLS). This indicates that the final sample was representative of the initial population enrolled in the study. 

**Table 2:** Sociodemographic variables stratified by groups: Dropouts vs. Completers.

Variables			Dropouts		Completers		p-value
			n=34	%	n=22	%	
Age (mean and SD)			(67.94 ± 6.47)		(68.64 ± 5.26)		0.465^*^
	Sex	Female	27	79.41	18	81.82	
		Male	7	20.59	4	18.18	0.825^†^
	Marital status	Widowed	6	17.65	2	9.09	
		Married/civil union	14	41.18	13	59.09	
		Separated/divorced	9	26.47	5	22.73	
		Single	5	14.71	2	9.09	0.567^†^
	Education	Primary complete	1	2.94	0	0.00	
		Secondary incomplete	1	2.94	0	0.00	
		Secondary complete	1	2.94	3	13.64	
		Higher incomplete	2	5.88	3	13.64	
		Higher complete	29	85.29	16	72.73	0.331
Education (mean and SD)			(18.67 ± 8.84)		(15.90 ± 3.52)		0.098^*^
	Retired or pensioner?	Yes	33	97.06	20	90.91	
		No	1	2.94	2	9.09	0.318^†^
	Who do you live with?	Alone	11	32.35	7	31.82	
		With partner only	11	32.35	9	40.91	
		With partner and your children	4	11.76	3	13.64	
		With your children	5	14.71	2	9.09	
		Your children live with you	2	5.88	0	0.00	
		Others live with you	1	2.94	0	0.00	
		With partner, children and grandchildren	0	0.00	1	4.55	0.533^†^

Abbreviation: SD, standard deviation.

* Mann-Whitney test;

† χ^2^ test.

**Table 3:** Descriptive statistics of applied scales stratified by groups: Dropouts vs. Completers.

Variables	Group	n	Mean	SD	Minimum	Median	Maximum	p-value
CFI Total T0	Dropouts	34	4.12	3.56	0.00	3.00	13.50	0.926
CFI Total T1	Completers	22	4.00	2.93	0.50	3.25	11.00	
GDS Total T0	Dropouts	34	3.88	3.46	0.00	2.00	11.00	0.291
GDS Total T1	Completers	22	3.05	3.44	0.00	2.00	13.00	
GAI Total T0	Dropouts	34	6.59	5.31	0.00	6.00	16.00	0.205
GAI Total T1	Completers	22	4.77	5.25	0.00	3.00	18.00	
SWLS T0 Total	Dropouts	34	7.53	1.38	3.00	8.00	10.00	0.725
SWLS T1 Total	Completers	22	7.68	1.29	5.00	8.00	10.00	

Abbreviations: CFI, Cognitive Function Instrument; GDS, Geriatric Depression Scale; GAI, Geriatric Anxiety Inventory; SWLS, Satisfaction With Life Scale. Note: p-value refers to Mann-Whitney test.

 Regarding the pre- and post-intervention analyses, the results showed a reduction in mean scores on the CFI (4.07 to 3.07), the GDS (3.55 to 2.32) and GAI (5.88 to 3.95) after the intervention, indicating improvements in perceived subjective cognitive decline, depressive symptoms, and symptoms of anxiety, respectively. The Wilcoxon test revealed a statistically significant difference in CFI scores (p=0.005) and GDS scores (p=0.046), suggesting the *Mentes Ativas* program may have promoted beneficial effects for these domains. The SWLS increased from 7.59 to 7.95, although this was not statistically significant (p=0.132), showing that perceived QoL remained essentially stable, albeit with a slight tendency for improvement (Table 4). 

**Table 4:** Descriptive statistics of scales applied.

Variables	n	Mean	SD	Minimum	Median	Maximum	p-value
CFI Total T0	56	4.07	3.30	0.00	3.00	13.50	0.005
CFI Total T1	22	3.07	2.75	0.00	2.50	12.00	
GDS Total T0	56	3.55	3.45	0.00	2.00	13.00	0.046
GDS Total T1	22	2.32	2.97	0.00	1.50	12.00	
GAI Total T0	56	5.88	5.31	0.00	5.00	18.00	0.109
GAI Total T1	22	3.95	4.99	0.00	1.50	18.00	
SWLS T0 Total	56	7.59	1.33	3.00	8.00	10.00	0.132
SWLS T1 Total	22	7.95	1.29	5.00	8.00	10.00	

Abbreviations: CFI, Cognitive Function Instrument; GDS, Geriatric Depression Scale; GAI, Geriatric Anxiety Inventory; SWLS, Satisfaction With Life Scale. Note: T0, Pre-test; T1, Post-test. p-value refers to Wilcoxon test.

 The correlation results reveal some significant associations. CFI scores at T0 showed a strong correlation with scores on the GDS (p=0.595; p<0.001) and the GAI (p=0.530; p<0.001), and a negative correlation with SWLS score (p=-0.471; p<0.001), suggesting that greater perceived subjective cognitive decline correlated with depressive and anxious symptoms, and with lower satisfaction with life. Similar relationships with the CFI at T1 were observed, strengthening the consistency of findings. In addition, performance on the GDS and GAI exhibited strong correlation with each other, both at T0 and T1 (p<0.001), confirming a positive correlation between depressive and anxious symptoms. There was also a significant negative correlation between GDS and SWLS, indicating higher levels of depression were associated with lower satisfaction with life. No statistically significant correlation of age and education with the other variables was found, although age showed a tendency toward correlation with CFI_T1 and GDS_T1 (Table 5). 

**Table 5:** Spearman correlation between continuous and ordinal variables.

Variable 1	Variable 2	rho	p-value
Age (years)	Education (years)	-0.203	0.142
	CFI_Total_T0	0.182	0.180
	CFI_Total_T1	0.231	0.300
	GDS_Total_T0	0.075	0.583
	GDS_Total_T1	0.327	0.138
	GAI_Total_T0	0.062	0.647
	GAI_Total_T1	-0.071	0.753
	SWLS_T0_Total	-0.124	0.361
	SWLS_T1_Total	-0.226	0.312
How many years of formal study do you have?	CFI_Total_T0	-0.072	0.603
	CFI_Total_T1	-0.035	0.879
	GDS_Total_T0	-0.116	0.402
	GDS_Total_T1	-0.213	0.353
	GAI_Total_T0	-0.006	0.964
	GAI_Total_T1	-0.201	0.383
	SWLS_T0_Total	-0.086	0.536
	SWLS_T1_Total	-0.138	0.550
CFI_Total_T0	CFI_Total_T1	0.804	<0.001
	GDS_Total_T0	0.595	<0.001
	GDS_Total_T1	0.582	<0.001
	GAI_Total_T0	0.530	<0.001
	GAI_Total_T1	0.667	<0.001
	SWLS_T0_Total	-0.471	<0.001
	SWLS_T1_Total	-0.301	0.174
CFI_Total_T1	GDS_Total_T0	0.528	0.011
	GDS_Total_T1	0.528	0.012
	GAI_Total_T0	0.430	0.046
	GAI_Total_T1	0.489	0.021
	SWLS_T0_Total	-0.195	0.383
	SWLS_T1_Total	-0.302	0.171
GDS_Total_T0	GDS_Total_T1	0.845	<0.001
	GAI_Total_T0	0.732	<0.001
	GAI_Total_T1	0.722	<0.001
	SWLS_T0_Total	-0.559	<0.001
	SWLS_T1_Total	-0.500	0.018
GDS_Total_T1	GAI_Total_T0	0.798	<0.001
	GAI_Total_T1	0.699	<0.001
	SWLS_T0_Total	-0.588	0.004
	SWLS_T1_Total	-0.715	<0.001
GAI_Total_T0	GAI_Total_T1	0.869	<0.001
	SWLS_T0_Total	-0.486	<0.001
	SWLS_T1_Total	-0.424	0.049
GAI_Total_T1	SWLS_T0_Total	-0.343	0.118
	SWLS_T1_Total	-0.386	0.076
SWLS_T0_Total	SWLS_T1_Total	0.777	<0.001

Abbreviations: CFI, Cognitive Function Instrument; GDS, Geriatric Depression Scale; GAI, Geriatric Anxiety Inventory; SWLS, Satisfaction With Life Scale. Note: T0: Pre-test. T1: Post-test.

## DISCUSSION

 The objective of the present study was to assess the efficacy of a remote multi-component cognitive stimulation program in cognitively healthy older adults who participated in the *Mentes Ativas* intervention of the USP60+ program of the EACH-USP. The results showed a significant reduction in perceived subjective cognitive decline and in depressive symptoms after the six-month intervention. There was also a reduction in anxious symptoms and a slight improvement in the life satisfaction variable, although this was not statistically significant. 

 Moreover, the results of the correlation analyses revealed a statistically significant association between higher scores for perceived subjective cognitive decline and greater levels of depressive and anxious symptoms, and also with lower life satisfaction, at assessments T0 and T1. 

 SCD is an increasingly important clinical construct given its association with higher risk of progression to MCI and dementia^8^ . Although the participants in the present study had no objective cognitive deficits, the self-reported perceived improvement may indicate higher confidence in cognitive abilities, as well as greater engagement and motivation in everyday activities^23^ . 

 The literature shows that SCD may be associated with emotional factors such as depression and anxiety, a relationship corroborated by the correlations found in this study. The hypothesis that improved SCD stems, at least in part, from a reduction in affective symptoms was reported in previous studies showing an interdependence between psychological wellbeing and cognitive perception^24,25^. Additionally, interventions for stimulating working memory, as applied in the program investigated, have shown benefits in promoting cognitive reserve and reducing subjective cognitive complaints. Evidence in the study by Borella et al.^26^ investigating the effects of working memory training in older adults, including oldest-old individuals, further support these findings. In their study, the training group had better performance than the active control group on tasks assessing everyday functioning, both at post-test and follow-up. 

 The study by Lindert et al.^27^ , analyzing longitudinal data from the Mid-Life in The United States Biomarker study, estimated the effects of symptoms of depression, anxiety and anger on cognition, with an emphasis on episodic memory and executive functions. The authors found a correlation between these symptoms and cognitive domains assessed in a longitudinal follow-up of young and older adults. More specifically, anxiety was found to negatively impact both of these cognitive domains in women and men. The findings confirm that impaired emotional aspects contribute to cognitive alterations over time and may lead to both objective and subjective cognitive decline. 

 Conversely, a reduction in negative emotional symptoms, as observed in the current analyses, can act as a mediator in improving perceived cognition. Evidence reported in the literature suggests that an improved emotional state in older individuals can promote a greater sense of control and engagement in cognitive and functional activities which, in turn, influence cognitive performance^10,28^. It is worth noting that the participants in this study presented low baseline levels of anxiety and depression, as the exclusion criteria ruled out major affective disorders. However, even within this subclinical range, significant reductions in GDS and GAI scores were observed. This suggests that the intervention may act by reducing the subclinical ‘affective load’, which directly influences the self-perception of cognition. This finding reinforces the relevance of addressing minor affective symptoms to improve subjective cognitive complaints in healthy older adults. 

 While the improvement in the life satisfaction variable was not statistically significant, scores on the SWLS were higher after the intervention. Hence, this outcome suggests that cognitive stimulation can promote gains, albeit modest, in terms of subjective wellbeing among cognitively healthy older adults. Overall satisfaction with life constitutes one of the key aspects of psychological wellbeing and is associated with perceived autonomy, social engagement, functioning and self-esteem^29^ . 

 In a study of older adults, Serra and Silva^30^ showed that greater life satisfaction was associated with better cognitive functioning and lower rate of subjective health complaints, suggesting a bi-directional association between perceived cognition and wellbeing. Corroborating these results, Yazdani and Siedlecki^31^ showed a relationship between objective cognition (for episodic memory, perceptual speed and reasoning ability) and subjective wellbeing (including life satisfaction and both positive and negative affect). The authors held that this relationship is mediated by factors including self-rated health, emotional stability and personality traits, such as conscientiousness^31^ . These outcomes highlight the importance of incorporating mental health indicators in assessments of the efficacy of cognitive intervention programs. 

 One of the main features differentiating the present study is its remote cognitive stimulation program using a digital online platform involving synchronous sessions. The level of participant adherence serves as a measure of the feasibility and acceptability of the program, where 22 participants attended at least 80% of the sessions. 

 Previous studies demonstrate that online cognitive interventions can be as effective as in-person training, provided they are adapted to the specific characteristics of the target sample^13,14^. The *Mentes Activas* program proved compatible with the technological abilities of the participants, with users receiving the necessary support to access the digital platform. The structured protocol of sessions any also have favored participant engagement, a feature that can be replicated by other research groups. 

 Some studies have shown maintenance of the effects of computerized and remote interventions over time (3–8 months) in healthy older adults^24,25^. Other investigations have demonstrated that remote cognitive stimulation can promote gains in functioning, directly assessed for instrumental activities of daily living, and may also reduce depressive symptoms, indicating a transfer effect of training to other domains besides cognition^32,33^. 

 There is a consensus in this investigative field that blind randomized controlled trials are the best option for assessing the effects of a cognitive intervention, and more recent studies have adopted these strategies as a means of enhancing their methodological rigour^33-35^. 

 Furthermore, the format of the *Mentes Ativas* program highlights the importance of early cognitive stimulation in the context of preclinical diagnosis and health promotion. Group-based online interventions represent a scalable, low-cost strategy for Primary Care, allowing non-specialists to facilitate cognitive stimulation. This approach overcomes geographic barriers and expands access to preventive strategies for healthy aging, reducing the burden on specialized tertiary centers. 

 This study has some limitations, such as lack of a control group, precluding any investigation of the influence of external factors on results. Another limitation was the absence of objective cognitive outcome measures at pre- and post-testing. Although the Braztel-MMSE was used for screening purposes at baseline, a comprehensive neuropsychological battery was not administered to evaluate objective cognitive changes. Additionally, self-report assessment scales were employed, possibly introducing response bias, such as social desirability or overestimation of subjective perception. 

 In summary, despite these limitations, this study showed that a remote cognitive stimulation program can promote gains for psychosocial variables in cognitively unimpaired older adults, most notably in the form of a reduction in perceived subjective cognitive decline and depressive symptoms, besides an increase in overall life satisfaction. 

 Future studies should include control groups, a more representative sample, and longitudinal follow-ups, to confirm the representativeness and maintenance of results over time. Lastly, studies on cognitive interventions that assess outcomes using neuroimaging techniques and incorporate digital cognitive training games represent a promising avenue of research. 

## Data Availability

The datasets generated and/or analyzed during the current study are available from the corresponding author upon reasonable request.
